# The mutation L69P in the PAS domain of the hERG potassium channel results in LQTS by trafficking deficiency

**DOI:** 10.1080/19336950.2020.1751522

**Published:** 2020-04-17

**Authors:** Tina Jenewein, Scott A. Kanner, Daniel Bauer, Brigitte Hertel, Henry M. Colecraft, Anna Moroni, Gerhard Thiel, Silke Kauferstein

**Affiliations:** a Institute of Legal Medicine, University of Frankfurt, Frankfurt Am Main, Germany; b Department of Physiology and Cellular Biophysics, College of Physicians and Surgeons, Columbia University, New York, NY, USA; c Computational Biology and Simulation Group, Department of Biology, Technische Universita ¨t Darmstadt, Darmstadt, Germany; d Department of Biology, Technische Universität Darmstadt, Darmstadt, Germany; e Department of Biosciences and CNR IBF-Mi, University of Milano, Milano, Italy

**Keywords:** LQT syndrome, hERG channel, dominant negative mutant, PAS domain, trafficking deficiency

## Abstract

The congenital long QT syndrome (LQTS) is a cardiac disorder characterized by a prolonged QT interval on the electrocardiogram and an increased susceptibility to ventricular arrhythmias and sudden cardiac death. A frequent cause for LQTS is mutations in the *KCNH2* gene (also known as the *human ether-a-go-go-related gene* or *hERG*), which reduce or modulate the potassium current I_Kr_ and hence alter cardiac repolarization. In a patient with a clinically diagnosed LQTS, we identified the mutation L69P in the N-terminal PAS (Per-Arnt-Sim) domain of hERG. Functional expression in HEK293 cells shows that a homotetrameric hERG channel reconstituted with only mutant subunits exhibits a drastically reduced surface expression of the channel protein thus leading to a diminished hERG current. Unlike many other mutations in the hERG-PAS domain the negative impact of the L69P substitution cannot be rescued by facilitated protein folding at a lower incubation temperature. Further, co-expression of wt and mutant monomers does not restore either wt like surface expression or the full hERG current. These results indicate L69P is a dominant negative mutation, with deficits which most likely occurs at the level of protein folding and subsequently inhibits trafficking to the plasma membrane. The functional deficits of the mutant channel support the clinical diagnosis of a LQTS.

## Introduction

The *KCNH2* or *hERG* (*human ether-a-go-go-related gene*) gene encodes the α-subunit of voltage-gated potassium channel Kv11.1, which conducts the rapidly activating delayed rectifier potassium current (I_Kr_). In the heart, hERG plays an important role in repolarization of the cardiac action potential and in the suppression of arrhythmic events caused by premature stimuli [1, 2, 3.] The hERG channel is a tetrameric protein, with each subunit containing voltage sensor (S1-4) and pore domains (S5-6) [4] as well as extended cytoplasmic domains. The latter harbors a functionally important Per-Arnt-Sim (PAS) domain in the amino-terminal [5], and a cyclic-nucleotide-binding homology domain (CNBD) in the carboxy terminus [6]. Loss of hERG channel function due to inherited mutations is a frequent cause of the long QT syndrome type 2 (congenital LQTS), which is characterized by a delayed ventricular repolarization (prolonged QT interval on the ECG) and an increased risk for fatal arrhythmias and sudden cardiac death (SCD) [7]. In addition, loss of hERG channel functionality can also be caused by off-target drug effects (acquired LQTS) [8].

Over 300 putative LQTS-causing missense mutations have been identified, but only half of them have been functionally studied in heterologous expression systems [9,10]. Most of the characterized missense mutations result in a reduction of the repolarizing outward potassium current (I_Kr_) and generate a prolonged repolarization of the cardiac action potential. The dominant molecular mechanisms by which *KCNH2* mutations cause a loss of hERG channel function are a modulation of channel gating and a reduction of intracellular transport or trafficking of the channel protein to the plasma membrane [3]. Recently a comprehensive study [9] showed that 88% of *KCNH2* missense mutations in the PAS domain, CNB domain, and pore domain produce trafficking deficient hERG channels. Additionally, this study showed that 70% of pore domain mutations produce a dominant negative effect, whereas PAS and CNB domain mutations did not [9].

In the present work, we identified a missense mutation in the *KCNH2* gene in a case of LQTS. The mutation is located in the N-terminal PAS domain of the hERG channel and leads to a non-conservative amino acid substitution L69P. To test whether the L69P mutation and two experimentally constructed variants at the same position have an impact on channel function, we performed patch-clamp measurements in HEK293 cells. Furthermore, we examined the trafficking of the hERG-L69P channel by imaging using intact HEK293 cells as well as isolated membrane patches. The data show that the mutation in the PAS domain has a dominant negative effect on the trafficking of the channel to the plasma membrane. This effect is not alleviated by incubating cells at a reduced growth temperature (27°C), a condition which is known to facilitate protein folding of some hERG channel mutants.

## Material and methods

### Clinical characteristics

A 35-year-old woman was suffering from dizziness and tachycardia in resting periods after physical stress. A complete cardiological examination including detailed anamnesis, a 12-lead electrocardiogram, exercise-testing, 24 h-holter monitoring, and transthoracic echocardiography was performed. Standard criteria for the diagnosis of LQT Syndrome (LQTS) were applied.

### Genetic analysis

The study was approved by the local ethic committee (E84/06). After receiving the informed consent of the patient, a blood sample for genetic investigations was taken. Targeted mutational analysis of the major ion-channel genes was performed as reported previously [11]. An additional evaluation of mutation pathogenicity was performed using the sequence-based programs PolyPhen-2 (Polymorphism Phenotyping v2), SIFT (Sorts Intolerant From Tolerant) and KvSNP (Single Nucleotide Polymorphism Prediction of Kv channels).

### Mutagenesis

QuickChange®II XL Site-Directed Mutagenesis Kit (Stratagene, Agilent Technologies) was used to introduce the *KCNH2*-L69P, *KCNH2*-L69A, and *KCNH2*-L69D mutations into the pEGFP-N2 vector with hERG *(KCNH2)* wild type. All constructs were verified by sequencing.

### Patch-clamp recordings

Human Embryonic Kidney (HEK293) cells were cultured and transiently transfected with 1 µg plasmid DNA using Gene Juice (Novagen, Merck, Darmstadt) or Turbofect (Thermo Fischer Scientifics, Waltham, MA, USA) according to the manufacturer’s protocol. One day after transfection the cells were isolated and transferred to new 35 mm plates in different concentrations. For confocal laser scanning microscopy (CLSM) the HEK293 cells were grown and transfected as mentioned above but on 25 mm round glass coverslips. Plasma membrane preparation was performed according to [12]. Patch clamp recordings were performed 48 h after transfection with an EPC-9 amplifier (HEKA, Lambrecht, Germany) in the whole-cell configuration using borosilicate glass pipettes (Tube capillary, Melting point, 1,5–1,8 mm, Kimble Chase, Gerresheimer, Vineland, USA) with a tip resistance of 2–5 MΩ. The extracellular bath solution contained 140 mM NaCl, 5.6 mM KCl, 1.2 mM MgCl_2_, 2.6 mM CaCl_2_ and 10 mM HEPES (pH 7,4, adjusted with NaOH). The internal pipette solution contained 140 mM KCl, 10 mM EGTA, 5 mM MgCl_2_, 2 mM CaCl_2_, 5 mM HEPES (pH 7.2, adjusted with KOH) and 5 mM ATP. All currents were recorded at room temperature. The voltage protocols are presented in the respective Figure legends.

### Confocal laser scanning microscopy (CLSM)

Confocal images of HEK293 cells expressing GFP tagged hERG channel protein were obtained 24–48 h after transfection on a Leica TCS SP5 II Confocal System equipped with an argon laser. The fluorescent marker Cellmask™ DeepRed (Invitrogen GmbH, Karlsruhe, Germany) was used for staining the plasma membrane of isolated membrane patches. Image acquisition was made with the HCX PL APO CS 100 × 1.44 OIL UV objective. The images were analyzed using the LAS AF software version 2.60 (Leica Microsystems CMS GmbH, Heidelberg, Germany).

### FACS analysis

For surface labeling experiments, the BBS-hERG-YFP construct was utilized as previously described [13,14], with the 13-residue bungarotoxin-binding site (BBS) introduced in the extracellular S3–S4 loop of hERG. Cell surface and total ion-channel pools were assayed by flow cytometry in live, transfected HEK293 cells [13,15]. Briefly, 48 h post-transfection, cells cultured in 12-well plates gently washed with ice cold PBS containing Ca^2+^ and Mg^2+^ (in mM: 0.9 CaCl_2_, 0.49 MgCl_2_, pH 7.4), and then incubated for 30 min in blocking medium (DMEM with 3% BSA) at 4ºC. HEK293 cells were then incubated with 1 µM Alexa Fluor 647 conjugated alpha-bungarotoxin (BTX-647; Life Technologies) in DMEM/3% BSA on a rocker at 4ºC for 1 h, followed by washing three times with PBS (containing Ca^2+^ and Mg^2+^). Cells were gently harvested in Ca^2+^-free PBS, and assayed by flow cytometry using a BD LSRII Cell Analyzer (BD Biosciences, San Jose, CA, USA). YFP-tagged channels were excited at 488 nm, and Alexa Fluor 647 was excited at 633 nm.

### Molecular dynamics (MD) simulations

The crystal structure of the N-terminal domain of hERG (PDB: 1BYW) was used for molecular dynamics simulations [5]. The L69P mutation was introduced with Pymol 2.2.0 [16]. Simulations were performed using Gromacs 2019.1 in combination with the CHARMM36 m force field (July 2017 revision) [17–19]. The TIP3Ps water model was used and each simulation box contained 0.15 mol KCl. Temperature and Pressure were kept at 298 K and 1 bar using the V-rescale thermostat and Parrinello-Rahman barostat, respectively [20,21]. The integration step was 2 fs. Van der Waals forces were force-switched to zero between 0.8 nm and 1.2 nm. Electrostatics were represented using the PME method and switched to zero between 0.8 nm and 1.2 nm [22]. Bonds including H-atoms were kept constant using LINCS [23]. Each simulation box was minimized, equilibrated with position restrains on the protein and simulated for 250 ns.

### Data analysis

Data are expressed as mean ± standard deviation (SD) of n experiments. An unpaired student-t-test was used to evaluate the significance of differences in the relative fluorescence intensity between hERG-WT and hERG-L69P cells.

## Results

### Cardiological findings

**Figure 1.**
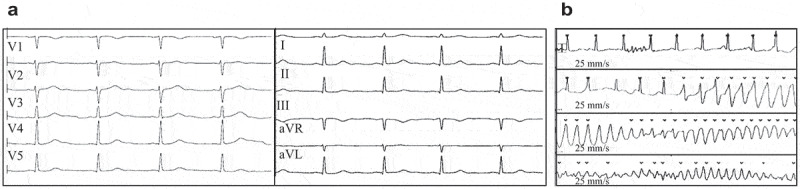
(a): 12-lead electrocardiogram of the patient revealing a prolonged corrected QT interval of 478 ms. Heart frequency: 63/min; PQ interval: 140 ms; QRS duration: 80 ms; QT: 468 ms; P-duration: 110 ms; RR/PP interval: 952/950 ms. (b) Electrocardiogram monitoring of the patient showed torsades de pointes.

The index patient was a 35-year-old women suffering from dizziness and syncopies. A cardiological examination revealed no morphological abnormalities (echocardiography, tilt-testing, exercise-testing, MRI, coronary angiography). Twelve leads ECG unveiled a prolonged corrected QT interval of 478 ms (Figure 1). During ECG monitoring a torsades de pointes tachycardia was documented. All these data pointing toward the diagnosis of an LQT syndrome (Schwartz Score > 4). A drug therapy with ß-blockers was started and an ICD was implanted.

### Mutation analysis

**Figure 2.**
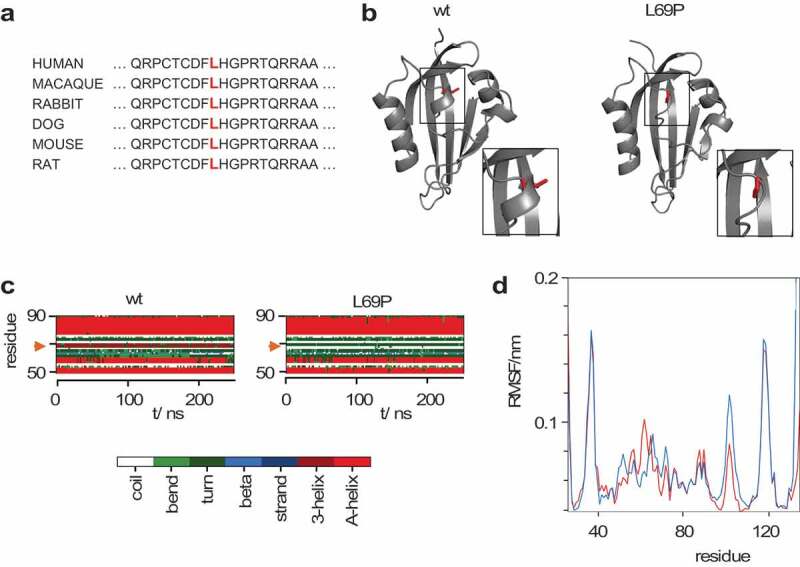
Structure of PAS domain in hERG channel and impact of L69P mutation. (a) Multiple sequence alignment of PAS domain ERG channels from different organisms. The part of the domain including L69 (red) in hERG is highly conserved. (b) Final snapshots of MD simulation of wt and L69P mutant. The regions boxed in the structures are magnified. The structure of the PAS domain is shown as a gray cartoon with residue 69 as red sticks. (c) DSSP plot of secondary structural elements in wt and L69P mutant for residues 50 to 90 in PAS domain. The 3-helix formed by residues 67–69 in the wt structure vanishes in the mutant; dark red band indicated by the arrow. (d) Root mean square fluctuation (RMSF) calculation for wt (red) *and* L69P mutant (blue).

Genetic analysis of LQTS related genes using direct sequencing revealed a heterozygous nucleotide change at position 206 from thymine to cytosine (CTG à CCG, rs199473665) in the exon spanning a region of KCNH2 gene. This substitution causes the amino acid substitution Pro for Leu at position 69. This residue within the N-terminal PAS domain of the hERG protein is highly conserved (Figure 2(a)) implying a crucial role for channel function and hence for maintaining normal cardiac activity. Indeed the *KCNH2* L69P mutation had already been previously associated with the Long-QT Syndrome (LQTS) [10] and was expected to be pathogenic according to bioinformatics prediction tools (Table 1). The *KCNH2* L69P mutation has however not been functionally characterized so far.

**Table 1.** Summary of pathogenicity prediction results.

Protein	CDS	PolyPhen-2	SIFT	KvSNP
p.L69P	c.206 T > C	Probably damaging (1.000)	Damaging	Disease (0.959)

Values in parentheses (0–1 for PolyPhen-2 and KvSNP) are a reliability index with larger values more reliable. CDS = coding sequences.

## Impact of L69P mutation on PAS domain structure

To better understand the impact of the L69P mutation on the structure of the PAS domain, we performed MD simulations on the wt and mutant PAS domains from the hERG channel. Figure 2(b,c) shows that the conserved Leu is positioned in a small 3-helix, which is stable over the entire time of simulation of the wt protein. In a mutant structure, in which Leu has been replaced by Pro, this 3-helix disintegrates during a 250 ns long simulation (Figure 2(b,c)). A scrutiny of the entire PAS domains shows that the impact of this mutation is local with little effect on the rest of the protein (Figure 1a supplement). The RMSF value (Figure 2(d)) and RMSD value (Figure 1b supplement) of the wt and the mutant structure are similar suggesting that the mutation does not destabilize the global structure of the PAS domain. A small sudden increase in the RMSD value of the mutant about 30 ns into the simulation (Figure 1b supplement) results from loss of secondary structure at the C-terminal end of the PAS domain (Figure 1a supplement). We consider this as an artifact of the simulation as it occurs at the edge of the isolated PAS domain and the deviation is still within the resolution of the crystal structure.

## Functional expression of hERG-WT and hERG mutants

**Figure 3.**
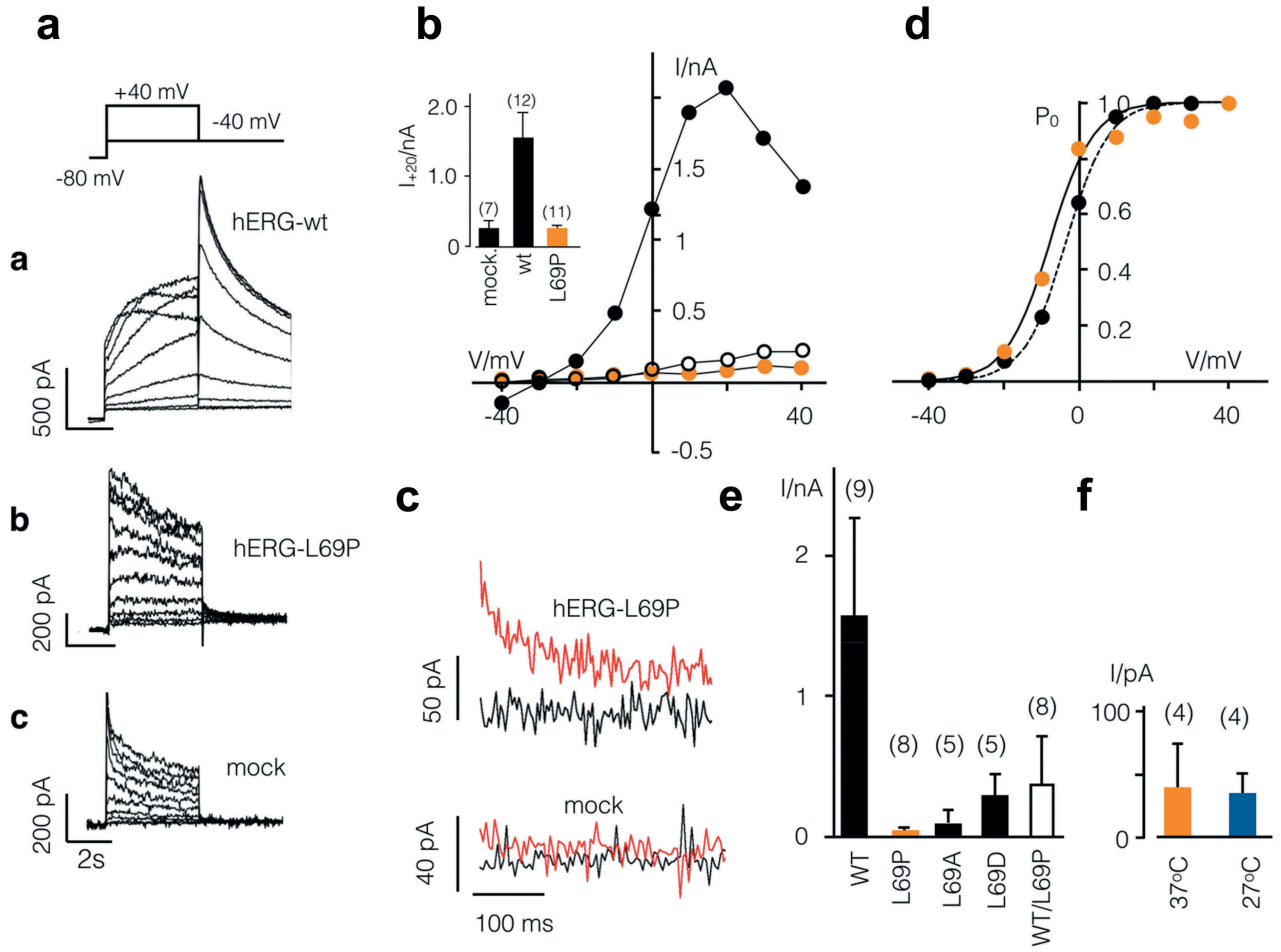
Electrical properties of hERG-wt and L69 mutants. (a) Voltage protocol (top) and representative currents recorded from HEK293 cells expressing hERG-wt (a), mutant hERG-L69P (b) or from mock transfected cell (c). (B) Steady-state I/V relations of currents from A; symbols in I/V relation cross-reference with symbols on current traces. *Inset*: Mean steady-state currents at +20 mV from mock transfected cells and cells expressing wt or L69P mutant (n = number of cells). (C) Magnification of tail currents from HEK293 cell expressing L69P mutant (top) and hERG-wt (bottom). Exemplary tail currents were measured at −40 mV after pre-pulse to +40 mV (red) or −40 mV (black). (d) Mean activation curves from tail currents of hERG-wt or L69P mutant. Data were fitted with a Boltzmann function: I/I_max_ = 1/(1+ exp((V_1/2_-V)/k)) (eqn. 1), where V_1/2_ is the half maximum activation voltage and k the slope factor. Fitting of the data yields mean values for half maximal activation (V_1/2_) of −4 mV for the wt (open bar) and −7 mV for the mutant (black bar). The slope factor is 4 for the wt and mutant. (e) Mean tail currents (±SD) from cells expressing hERG-wt, different mutations of residue L69 and from cells co-transfected with hERG-wt and hERG-L69P (WT/L69P) at 1:1 ratio. Tail currents were recorded as in A at −40 mV after pre-pulses to +40. (f) Tail currents of hERG-L69P mutant as in E from cells incubated at either 37°C or 27°C. Number of cells for different experiments are given in brackets.

To investigate the functional impact of the hERG-L69P mutation, wt and mutant channels with a C-terminally attached GFP were transiently expressed in HEK293 cells [24]. Whole-cell patch clamp recordings on GFP positive cells show the typical activation of the hERG-wt channel in response to different depolarizing steps (Figure 3(Aa)). Cells transfected with the wt channel exhibited large hERG type outward currents, which decreased at positive voltages > +20 mV due to an inherent inactivation. During a repolarizing pulse to −40 mV the channels recovered from inactivation; this transient opening generated the large tail currents characteristic for hERG. The hERG-L69P transfected cells only exhibited small currents (Figure 3(Ab)), which were not much different from those of mock-transfected cells (Figure 3(Ac,B)). Neither the currents nor the I/V relations from the mutant showed the negative conductance at positive voltages (Figure 3(a,b)). By contrast to controls, cells expressing hERG-L69P frequently exhibited a small but distinct tail current at −40 mV (Figure 3(c,e)). A plot of normalized activation curves from the tail currents of wt and mutant channel shows a similar voltage dependency (Figure 3(d)). Both activation curves can be fitted with a Boltzmann function:

(1) $$I/I_{max}=1/\left(1+exp\left(\left(V_{1/2}-V\right)/k\right)\right)$$

where V_1/2_ is the half-maximum activation voltage and k the slope factor.

A fit of the data yields similar V_1/2_ values and slope factors (k) for mutant and wt currents: wt: V_1/2_ = −4 ± 2 mV, k = 4.4 ± 0.2 mV; L69P: V_1/2_ = 7.5 ± 2 mV, k = 4.3 ± 0.2 mV. The results of these experiments suggest that the hERG-L69P mutant generates a functional channel with a low channel density or reduced unitary channel current in the plasma membrane.

It is well established that many hERG mutants with trafficking deficits can be rescued by incubating cells at low temperature; this treatment presumably supports a correct folding of the protein, which is otherwise compromised by the mutation [13,25–27]. To examine whether the L69P mutation can be rescued in this manner we repeated the same experiments as in Figure 3(Ab) with cells incubated at 27°C. The data in Figure 3(f) however show that the characteristic small tail currents of the mutant are not different from those measured in cells incubated at 37°C.

The replacement of the nonpolar amino acid leucine by the nonpolar, aliphatic amino acid proline seems to disturb the structural integrity of the PAS domain resulting in either a trafficking or a functional defect of the hERG channel, or both. To test if this disturbance by the mutation is specific to the unique structural impact of proline on protein folding [10] and polypeptide structure [28] we created two additional point mutations at position 69. The nonpolar leucine was therefore substituted with alanine (A) and aspartic acid (D). Alanine is also nonpolar but significantly smaller than leucine, whereas aspartic acid contains a negatively charged side chain. Patch clamp recordings revealed that also these mutations were not able to recapitulate the wt phenotype. About 50% of transfected HEK293 cells exhibited – unlike the L69A mutant – a hERG type current with a typical activation and deactivation at positive voltages followed by distinct tail currents (Figure 2a supplement). But the amplitude of these tail currents remained significantly smaller than those recorded in cells expressing the wt channel (Figure 3(e)); also the deactivation of the tail currents from the mutant channels is faster than in the wt channel (Figure 2a supplement). The results of these experiments show that the position L69 is generally sensitive to amino acid exchanges. The substitution of a Leu to Pro, which is found in the patient, however, is more severe than other amino acid substitutions.

To imitate the heterozygous situation typical in patients, we co-transfected HEK293 cells with both the L69P mutant and the wt-hERG channel in a 1:1 ratio. Co-expression of wt and mutant channel generated a large spectrum of current responses ranging from cells with no detectable currents to cells with typical hERG type currents (Figure 2b supplement). The mean amplitude of the tail currents from 8 cells is in this situation below 50% of that recorded in cells, which express the wt channel. The results of these experiments imply that the L69P mutation has a dominant negative effect on the current density of the hERG channel.

### The L69P mutant exhibits a reduced density in the plasma membrane

**Figure 4.**
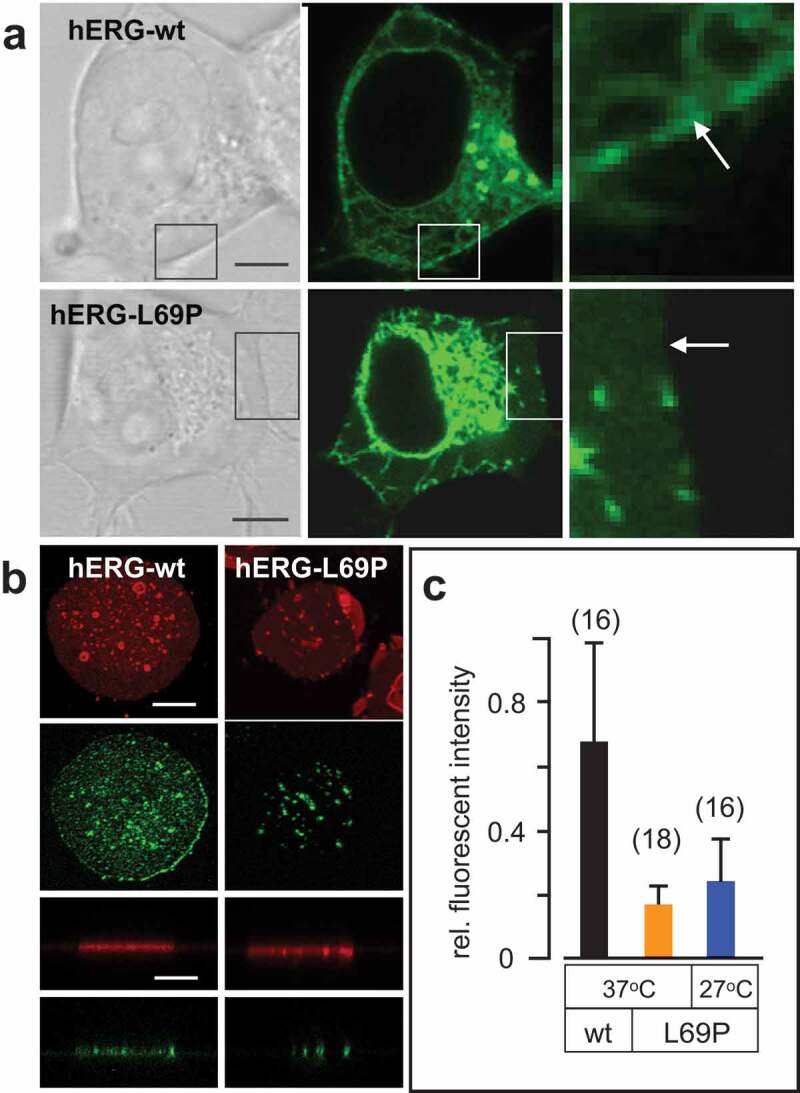
Impact of L69P mutation on protein synthesis and plasma membrane targeting. (a) Representative HEK292 cells expressing GFP tagged hERG-wt (top) or L69P mutant (bottom). Cells are show from left-to-right as bright field (left) and fluorescent images (central). The areas indicated by boxes are magnified in the right column. Arrows indicate the position of the plasma membrane. (b) Confocal image of isolated plasma membrane patches of HEK293 cells, which transiently express either the GFP tagged hERG wt (left column) or the L69P mutant (right column). The red channel shows fluorescently staining of membrane patches with CellMaskTM Deep Red, while the green channel reports fluorescence from GFP. The two lower rows show the red and green fluorescence of the respective membrane patches in a side view perspective. Note that the cells are fully decapitated leaving only isolated membrane patches. For a quantitative analysis, the fluorescence was measured from the side view images in regions of the red fluorescence and the respective area of the green channel. (c) Mean relative GFP fluorescence in membrane patches from HEK293 cells expressing hERG-wt or L69P mutant at 37°C or 27°C. Data were obtained from side view images as in B by dividing fluorescence intensity of green channel by respective value of the red channel. Number of cells in brackets. Scale bars 5 μm.

The results of the functional characterization suggest that the mutation reduces the number of active hERG channels in the plasma membrane. To further examine a potential defect in protein synthesis and/or trafficking we monitored a GFP construct by CLSM. Representative images of HEK293 cells expressing the hERG-WT and mutant channel L69P are depicted in Figure 4(a). Cell expressing the hERG-WT channel exhibited a clear GFP fluorescence in the plasma membrane. In contrast cells expressing the hERG mutants revealed a strong GFP fluorescence only in association with intracellular compartments. While the endoplasmatic reticulum with the perinuclear ring is strongly fluorescent the plasma membrane is mostly not labeled (Figure 4(a)).

In the next step, we examine the apparent difference in channel concentration in the plasma membrane between wt and mutant without any interference from intracellular compartments. Therefore, we isolated plasma membrane patches of HEK293 cells expressing hERG-WT and hERG-L69P as reported previously [12]. The images in Figure 4(b) show that after decapitation of the cell body only small patches of plasma membrane remain attached to the coverslips. These isolated membrane patches from cells expressing the wt or the L69P mutant contain GFP fluorescence (Figure 4(b)). Hence, both wt and mutant are positively targeted to the plasma membrane. To compare the relative GFP fluorescence of the isolated membranes the green fluorescence associated with these patch was measured. To account for different sizes of the isolated membrane patches the fluorescence intensity from the green channel was divided by the red fluorescence from the same area of interest; the red fluorescence from Cellmank^TM^ DeepRed provides in this case a relative measure of the membrane size. Figure 4(c) shows that hERG-L69P:GFP exhibits a significant lower fluorescence in the plasma membrane than the hERG-WT:GFP construct. The results of these experiments confirm the visual impression from images in Figure 4(a) in that the density of the mutant channel in the plasma membrane is lower than that of the wt channel. The imaging data are in agreement with the electrical recordings from Figure 3. The comparative analysis of confocal images from cells incubated at 37°C and 27°C furthermore shows that incubation at low temperature has a small positive effect on the trafficking of the channel to the plasma membrane. But in agreement with the electrical recordings it does not rescue the mutant phenotype. (Figure 3(f)).

**Figure 5.**
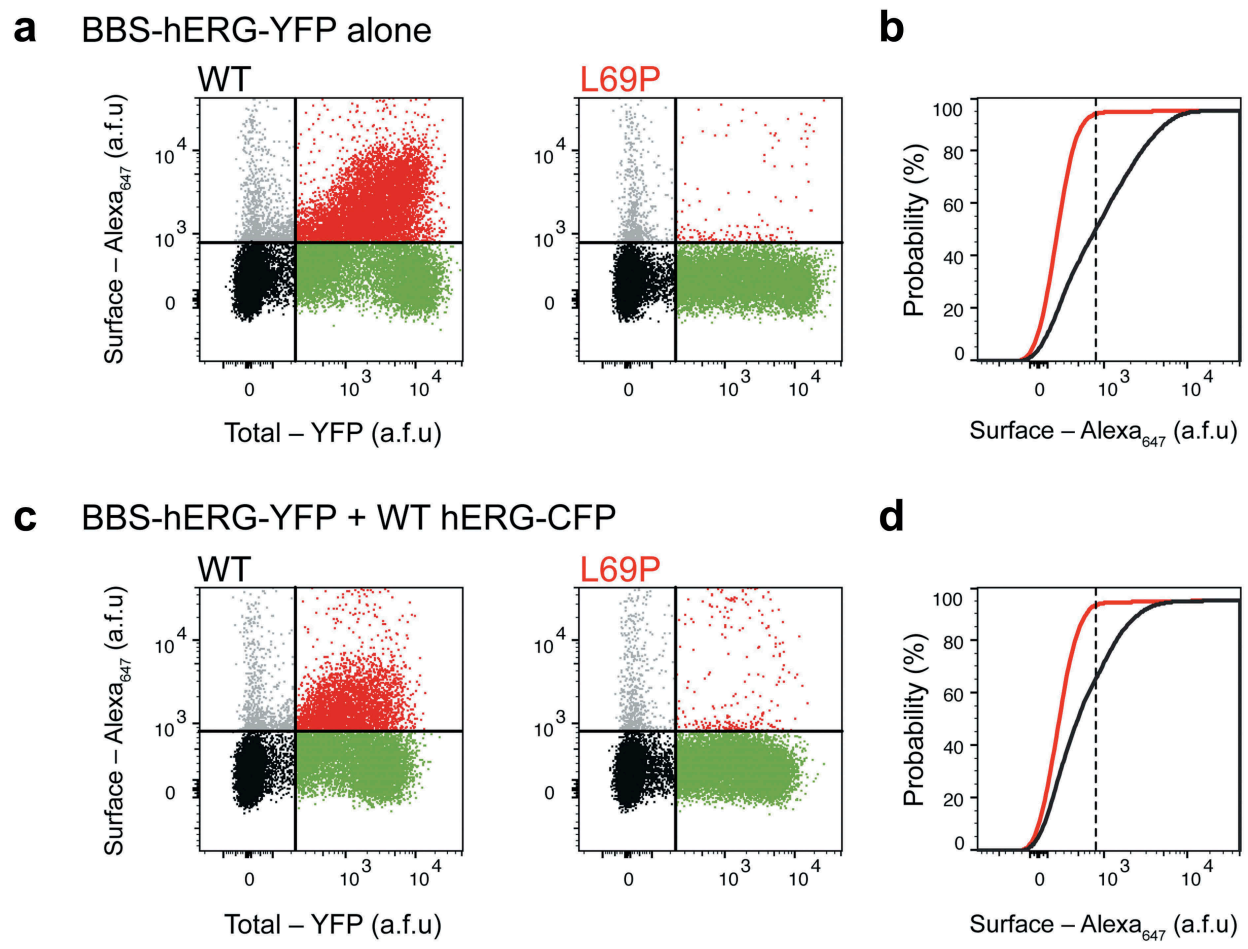
Flow cytometric analysis of surface expression with L69P mutants ± WT hERG channels. (a) Flow cytometry dot plot showing surface (BTX647 fluorescence) and total (YFP fluorescence) hERG expression in cells expressing BBS-hERG-YFP. Vertical and horizontal lines represent thresholds for YFP and BTX647-positive cells, respectively, based on analyses of single-color controls. Represented are YFP-positive cells with BTX647 signal above (red dots) or below threshold (green dots); BTX647-positive cells with YFP signal below threshold (gray dots); and untransfected cells (black dots). (b) Cumulative distribution histograms of Alexa_647_ fluorescence from flow cytometry analyses. Plot generated from population of YFP-positive cells (*n* ≥5000 cells per experiment). Dotted line is the threshold value for BTX_647_ signal. (c, d) Same experiments as in (a,b), but upon co-expression with WT hERG-CFP channels.

To directly assess differences in the surface expression of wt and the L69P mutant, a flow cytometric assay was performed as previously reported [13]. HEK293 cells were transfected with the BBS-hERG-YFP construct, engineered with a 13-residue high-affinity bungarotoxin binding site (BBS) extracellular tag for the efficient detection of surface channels in non-permeabilized cells with Alexa Fluor 647-conjugated bungarotoxin (BTX647). In addition, a C-terminal YFP tag enabled the simultaneous fluorescence detection of total hERG expression. Analysis by flow cytometry allowed for the evaluation of hERG surface/total expression in ~50,000 live cells, with single-cell resolution. Consistent with subcellular and electrophysiological analyses, cells expressing L69P BBS-hERG-YFP (i.e. YFP-positive cells) displayed a reduction in surface intensity (Figure 5(a), right) compared with wt BBS-hERG-YFP (Figure 5(a), left). This decrease in surface expression is represented by a left-ward shift in the population histogram of Alexa-647 intensity in cells expressing the L69P mutant (Figure 5(b)). Furthermore, co-expression of untagged wt hERG-CFP subunits with L69P BBS-hERG-YFP at a 1:1 ratio did not alter L69P mutant trafficking, consistent with the data from whole-cell recordings of the heterozygous condition (Figure 2(e)).

## Discussion

The present data confirm a previous assumption that the mutation L69P within the PAS domain of hERG can be the cause of an LQT syndrome [10]. Transient expression of the mutant channel or a mix of wt and mutant channel, which mimic the homozygous and heterozygous situations, respectively, cause a drastic reduction in the density of the channel protein in the plasma membrane of cells. This reduction in channel density is compatible with the suppression of hERG currents recorded in cells expressing the mutant alone or in combination with the wt channel. A similar strong reduction in hERG channel density with the concomitant reduction in hERG current has already been identified in the context of other hERG mutants as a cause of the LQT syndrome [29].

The combined electrophysiological recordings and cell biological experiments underscore a dominant negative effect of the L69P mutation on trafficking of the channel protein to the plasma membrane. The fact that the few mutant channels, which reach the plasma membrane, seem to exhibit the functional properties of the wt channel, suggests that the mutation *per se* may not substantively compromise channel gating. This interpretation is consistent with the finding from MD simulations that the mutation has no major effect on the structure of the PAS domain. It seems more likely that the mutation interferes with the folding of the nascent protein. This mutant polypeptide must then interact with the wt hERG subunits with the consequence that the tetrameric hERG channel is no longer efficiently sorted to its final destination.

This interpretation is in good agreement with the general picture of the hERG PAS domain as a hot spot for missense mutations that result in a decreased trafficking of the channel [30,31]. In line with the present results, recent data have already shown that the interaction between the N-cap amphipathic helix in the core of the PAS domain plays a critical role in stabilizing the isolated PAS domain [32]. The authors suggested that mutations in the PAS core, which disrupt this self-assembling structure, will lead to trafficking defects of the hERG channel. Furthermore, they demonstrated that channels lacking the PAS domain (∆2-135) traffic perfectly well. This implies that if the PAS domain is present, it must be intact and properly fold during translation.

The data show that the strong negative effect of the L69P mutation can be alleviated by substituting Pro in position 69 by other amino acids. The results of these experiments imply that position 69 is in general critical for a proper folding of the channel protein. The finding that Pro has a stronger negative impact than other amino acids is not surprising considering the strong structural impact of this amino acid on protein structures and on the kinetics of protein folding [10]. The L69P mutation is located at the C-terminal end of the single turn of this 3_10_ helix (Figure 2). The side chain of leucine 69 intrudes into the hydrophobic core of the PAS domain and is additionally stabilized by hydrogen bonds with the backbone carbonyl of Y99 and C66. The presence of proline at the C-terminal cap of 3_10_ helices is energetically disfavored and might induce distortions [33]. This assumption is confirmed by MD simulations, which show that Pro causes in the folded protein a disintegration of the 3-helix during a 250 ns long simulation. Proline is a unique amino acid in that the side chain is cyclized back on to the backbone amide position. As a general consequence proline is unable to function as a hydrogen bond donor and this can introduce kinks into α-helices [34].

Previous studies have shown that deficient trafficking of hERG channels with missense mutations in the PAS core, which are located next to amino acid position 69 (T65P, C66 G, F68 L and H70 R) can be improved by lowering the incubation temperature to 27°C [9,35]. A lower temperature presumably supports a correct folding of the protein. The present data show that a lower incubation temperature has no positive effect on the L69P mutant. This suggests that the substitution of Pro for Leu has a more severe structural impact on the fold of the protein, which is not compensated by a slower rate of protein synthesis.

In conclusion, our data show that a mutation in the PAS domain of the hERG channel is responsible for a dominant mechanisms of mutant protein actions in an LQT patient. The dominant nature of the mutant effect on channel trafficking is rather unusual considering that a large-scale analysis of hERG mutants associated dominant negative effects more frequently with mutations in the pore domain rather than in the PAS domain [9]. The functional and structural data are however not able to answer the questions on the specific steps of protein synthesis, folding and/or trafficking, which are corrupted by this mutation. Also, it remains elusive why the impact of other missense mutations in the direct vicinity of this mutation on folding can be corrected by low temperature while the L69P mutant remains unaffected by this treatment.
