# Supplementary figures and images for: The mutation L69P in the PAS domain of the hERG potassium channel results in LQTS by trafficking deficiency
Source: Channels (Austin). 2020 Apr 17;14(1):163–74. doi: 10.1080/19336950.2020.1751522 (PMC7188350; doi:10.1080/19336950.2020.1751522)

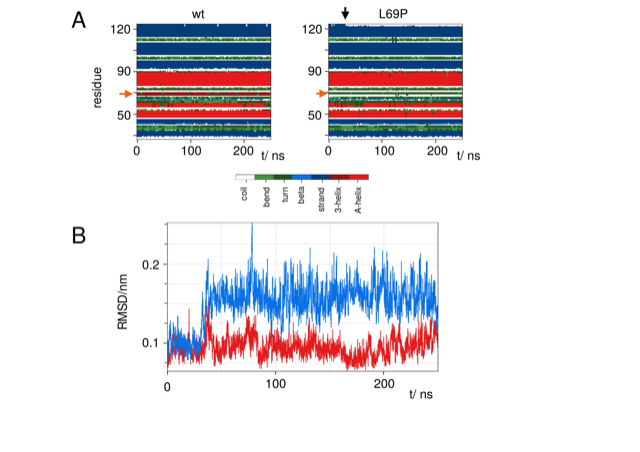

Supplement: Supplemental Material [file kchl-14-01-1751522-s001.zip › Fig_s1.png]

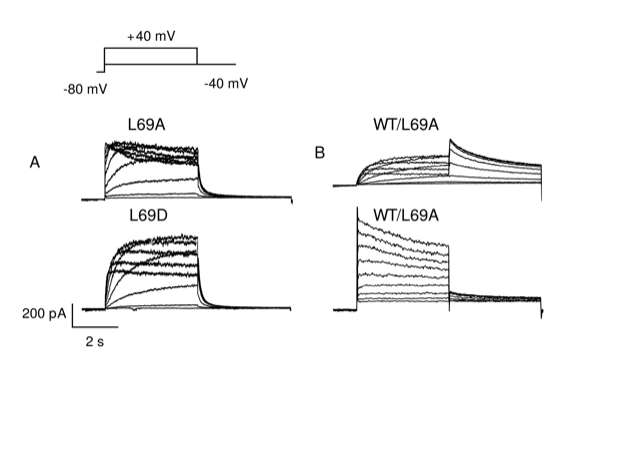

Supplement: Supplemental Material [file kchl-14-01-1751522-s001.zip › Fig_s2.png]
